# Survey of infection control measures and design of emergeny rooms in Quebec, Canada: an overview of the actual situation

**DOI:** 10.1186/1753-6561-5-S6-P265

**Published:** 2011-06-29

**Authors:** A-M Lowe, P Dolcé, B Baril, C Sauvé, D Goulet, A Fortin

**Affiliations:** 1 INSPQ, Montreal, Canada; 2 CSSS Rimouski, Rimouski, Canada; 3 Emergency department, Charles-Lemoyne Hosp., Greenfield Park, Montreal, Canada; 4 Emergency department, Ste-Justine Hosp., Montreal, Canada; 5 Inf. ctrl., CHUQ, Québec, Canada; 6 INSPQ, Québec, Canada

## Introduction / objectives

Emergency rooms (ER) are considered the "front door" of hospitals and are facing overcrowding, high intensity of cares, non-optimal design and a quick turnover of patients and personnel. Very few papers in the litterature discussed infection control (IC) measures in ER. This study evaluated the situation of IC in Quebec’s ER.

## Methods

An electronic survey covering design, hand hygiene, IC measures and housekeeping of ER was done using Survey Monkey software. The questionnaire was sent in September 2010 to IC practitioners of Quebec acute-care settings with >1000 admissions/year. Data were analyzed with Epi-Info 3.5.2.

## Results

The survey was completed by 63/89 (71%) hospitals. ER had a mean of 22 beds (range: 5-52), including 30,3% of single rooms. Airborne isolation rooms (AIR) were present in 87% of ER (range: 0-8 AIR). The ratio of ER that had a proportion of toilet/bed between 0 to 40% was 85%. Hand hygiene stations were located next to 78,5% of beds. Audits on hand hygiene compliance were performed in 35/63 (55,5%) of ER within the past two years. The compliance rate was <50% in 90,6% of ER. A designated area in the waiting room to cohort patients presenting with infectious diseases symptoms was present in 87,1% ER. Surveillance of MRSA and VRE were done in 90,4% of ER. An IC committee specific to ER was implemented in only 4,8% of ER. Dedicated housekeeping personnel were present in 76,2% of ER.

## Conclusion

In Québec’s ER, only 30% of beds were designed as single rooms and compliance to hand hygiene was low. More evidence-based data and guidelines are needed on IC in ER.

## Disclosure of interest

None declared.

